# Sex Differences in the Effect of Inflammation on Subjective Social Status: A Randomized Controlled Trial of Endotoxin in Healthy Young Adults

**DOI:** 10.3389/fpsyg.2019.02167

**Published:** 2019-10-01

**Authors:** Mona Moieni, Keely A. Muscatell, Ivana Jevtic, Elizabeth C. Breen, Michael R. Irwin, Naomi I. Eisenberger

**Affiliations:** ^1^Department of Psychology, University of California, Los Angeles, Los Angeles, CA, United States; ^2^Cousins Center for Psychoneuroimmunology, University of California, Los Angeles, Los Angeles, CA, United States; ^3^Department of Psychology and Neuroscience, University of North Carolina at Chapel Hill, Chapel Hill, NC, United States; ^4^UNC Lineberger Comprehensive Cancer Center, University of North Carolina at Chapel Hill, Chapel Hill, NC, United States

**Keywords:** inflammation, subjective social status, social behavior, endotoxin, sex

## Abstract

It has been established that inflammation leads to a variety of changes in social experience, but one area of social experience that has been overlooked is subjective social status. Furthermore, given sex differences in the relationship between inflammation and social status, males may be more sensitive to inflammation-induced changes in social status. However, no previous studies in humans have examined this possibility. In the present study, healthy young participants (*n* = 115) were randomly assigned to receive either endotoxin, an experimental inflammatory challenge, or placebo. Participants reported their subjective social status at baseline (prior to injection), and approximately 2 h later (time of peak inflammatory response for the endotoxin group). Results, using ANCOVA analyses, indicated that males exposed to endotoxin, but not females, reported lower levels of subjective social status at the peak of inflammatory response (vs. placebo). These results suggest that males may be more sensitive to the effects of inflammation in certain social domains, such as perceived social status.

**Clinical Trial Registration:** www.ClinicalTrials.gov, identifier NCT01671150.

## Introduction

During sickness, proinflammatory cytokines induce a constellation of symptoms known as “sickness behavior” (Kelley et al., 2003). In addition to physical symptoms associated with being sick—such as fatigue, pain, and loss of appetite—sickness behavior also involves changes in social behavior. This includes increases in sensitivity to negative social experiences, such as increases in feelings of social disconnection in humans and social withdrawal behavior in non-human animals, as well as increases in sensitivity to positive social experiences, such as increases in desires to be around close others in humans and social-clinging behavior in non-human animals (Eisenberger et al., 2017; Moieni and Eisenberger, 2018). These changes are thought to be evolutionarily adaptive; individuals in a heightened inflammatory state need to rest and recover, which may promote social withdrawal and increased sensitivity to negative social experience in order to conserve energy for recuperation. At the same time, these individuals are also are in a vulnerable state, which may promote increased sensitivity to positive social experience and social affiliative behavior to find allies who may aid them in this vulnerable state (Eisenberger et al., 2017; Moieni and Eisenberger, 2018).

One area that remains underexamined in the context of inflammatory-induced changes in social experience in humans is social status. In mice, experimental inflammatory changes can destabilize social hierarchies and disrupt social status-associated behavior, such as aggression (Cirulli et al., 1998; Cohn et al., 2012). Indeed, it has been suggested that one of the functions of inflammation may be to promote behavior that would indicate submissiveness, which could be adaptive in order to prevent further aggression in a time of vulnerability, such as during sickness (Kemeny, 2009). Although we are not aware of any studies examining the effects of experimental inflammation on social status in humans, correlational studies support a relationship between social status and inflammation, such that lower social status is associated with higher levels of inflammation (Muscatell et al., in press). Although it is possible (and often assumed) that these associations are driven by low status leading to higher inflammation, it is also possible that higher inflammation is leading to low status.

Interestingly, subjective social status, defined as an “individual’s perception of [their] own position in the social hierarchy” (Jackman and Jackman, 1973), is a unique predictor of health outcomes (Cundiff and Matthews, 2017; Zell et al., 2018) over and above objective status measures (e.g., income, education). For example, subjective social status is predictive of self-related health (Singh-Manoux et al., 2005), cardiovascular disease (Tang et al., 2016), mental health (Singh-Manoux et al., 2005), and mortality (Demakakos et al., 2018). However, subjective social status remains understudied in the context of inflammation (Muscatell et al., in press), particularly when considering how changes in inflammation may influence individuals’ subsequent perceptions of their social status.

Another important factor to examine in the context of social status and inflammation is sex. Social status is more relevant to males’ self-esteem and ability to secure a mate than females’ (Buss, 1989; Fischer and Mosquera, 2001; Zentner and Mitura, 2012; Freeman et al., 2016). Thus, given this heightened importance of social status for males, males may be more sensitive to inflammatory-induced changes in perceptions of social status. Interestingly, in humans, there is some relevant correlational data to support this notion; the relationship between subjective social status and inflammation has been found to be stronger in men than women (Freeman et al., 2016).

Given these findings, it follows that males may be particularly sensitive to inflammatory-induced changes in social status. However, no studies in humans have examined the effects of experimental inflammation on social status or possible sex differences in such changes. Thus, we sought to fill this gap in the literature with the present study by examining the effect of endotoxin on subjective social status using a previous dataset. We hypothesized endotoxin (vs. placebo) would lead to lower levels of subjective social status for males at the peak of inflammatory response, but not for females.

## Materials and Methods

### Participants and Procedures

One hundred and fifteen healthy participants (69 female; mean age: 24.2 ± 6.6 years) who met inclusion criteria (e.g., did not have chronic inflammatory disorders, Axis-I psychiatric disorders; met fMRI safety criteria for a separate component of the study) completed the study. Full exclusionary criteria, as well as full demographic information about this sample, can be found elsewhere (Moieni et al., 2015c). Of the 115 participants, 54 were randomized into the placebo condition (31 female) and 61 were randomized into the endotoxin condition (38 female). The flow of participants is available in the CONSORT diagram in the Supplementary Material of this paper.

Detailed descriptions of study procedures have already been reported (Moieni et al., 2015a, c), but are summarized here. None of the subjective social status data in this paper have been reported on previously; however, other results from the parent study have been published (Inagaki et al., 2015; Moieni et al., 2015a,b,c, 2019; Muscatell et al., 2016; Cho et al., 2017, 2019; Irwin et al., 2019). Thus, this is a secondary analysis of these data.

The study was conducted at the UCLA Clinical and Translational Research Center (CTRC); the study took place between March 2011 and August 2013. The study design was a randomized, double-blind, placebo-controlled design. Participants were randomly assigned to receive endotoxin or placebo ninety minutes after arriving to the CTRC. The low-dose endotoxin (0.8 ng/kg of body weight) was derived from *E. coli* (*E. coli* group O:113: BB-IND 12948 to MI) and provided by the National Institutes of Health Clinical Center (Suffredini and O’Grady, 1999). The placebo was the same volume of 0.9% saline. Both were administered by a nurse as an intravenous bolus. Levels of proinflammatory cytokines were assessed through hourly blood draws, starting at baseline prior to endotoxin or placebo administration. Blood draws were then taken approximately every hour over a total time of six and a half hours after participants received endotoxin or placebo.

All subjects provided written consent before participating. All procedures were approved by the UCLA Human Subjects Protection Committee. The study was registered as a Clinical Trial (#NCT01671150).

### Plasma Levels of Cytokines

Plasma tumor necrosis factor (TNF)-α and interleukin (IL)-6 concentrations were determined using a high sensitivity bead-based multiplex immunoassay (Performance High Sensitivity Human Cytokine, R&D Systems, Minneapolis, MN, United States), as previously described (Moieni et al., 2015a,b,c). The full cytokine profile for participants from this exact study is available in other publications (Moieni et al., 2015b,c).

### Subjective Social Status

Subjective social status was assessed at baseline (*T0*) and approximately 2 h post-endotoxin/placebo administration (*T2*), the peak of the inflammatory response for the endotoxin group, using the community version of the MacArthur Scale of Subjective Social Status (Adler et al., 2000; Adler and Stewart, 2007). This oft-used measure presents participants with a pictorial depiction of a ladder and asks them to denote which rung of the ladder they feel they are on, relative to others in their community. The instructions read: “Think of this ladder as representing where people stand in their communities. People define community in different ways; please define it in whatever way is most meaningful to you. At the top of the ladder are people who have the highest standing in their community. At the bottom are people who have the lowest standing. Where would you place yourself on the ladder? Please place a large ‘X’ on the rung where you feel you stand *right now*, relative to other people in your community.” (Scores can range from 1 to 10). Note that these instructions are identical to the original version of the measure, except for the timing participants are asked to evaluate. In the original, participants are asked to reflect on their standing “at this time in your life”; in order to capture changes in response to endotoxin, this was changed in the present study to “right now.” Descriptive statistics of subjective social status scores at *T0* and *T2* are provided in the Supplementary Material.

### Statistical Analyses

Full details of the overall effects of endotoxin on cytokine levels are reported elsewhere (Moieni et al., 2015b,c). Because cytokine values were not normally distributed, values were natural log-transformed, and due to known effects of BMI on cytokines, we controlled for BMI in all cytokine analyses.

For the current analyses, we focused on the baseline timepoint (prior to injection; *T0*) and the timepoint associated with the peak of inflammatory response for the endotoxin group (approximately 2 h post-injection; *T2*). As noted above, these are also the only two timepoints at which subjective social status was measured. To assess between-group differences in the effect of endotoxin vs. placebo on cytokines and subjective social status, we used a standard statistical software program (SPSS 25.0) to conduct analyses of covariance (ANCOVA). These analyses tested condition (endotoxin vs. placebo) effects at *T2* controlling for *T0*, as well as sex (male vs. female) by condition interactions. Follow-up analyses for significant interactions were conducted within each sex to examine differences between conditions at *T2*, controlling for *T0* (e.g., subjective social status within males between endotoxin and placebo at *T2*, controlling for *T0* values). Effect sizes were calculated for subjective social status effects.

To further probe these relationships, we also looked at correlations between changes from *T0* to *T2* in cytokines and subjective social status scores among subjects in the whole endotoxin sample, as well as for males and females separately.

Because we have previously shown effects of endotoxin on other socioemotional responses (i.e., self-reported depressed mood and self-reported feelings of social disconnection; Moieni et al., 2015c), which may be relevant to subjective social status, all analyses with subjective social status as an outcome included these socioemotional variables (i.e., depressed mood and feelings of social disconnection at *T2*) as covariates.

## Results

### Inflammatory Responses

As reported previously (Moieni et al., 2015b,c), endotoxin (vs. placebo) led to significant increases in IL-6 and TNF-α from *T0* to *T2* (*p*’s < 0.001). There were no sex differences in the magnitude of the cytokine response (*p*’s > 0.8).

### Subjective Social Status

First, we tested the effects of endotoxin (vs. placebo) on subjective social status for all participants. We found no significant overall condition differences in self reports of subjective social status at *T2* [F(1,109) = 0.35, *p* = 0.56], indicating no main effect of endotoxin (vs. placebo) on subjective social status.

**FIGURE 1:**
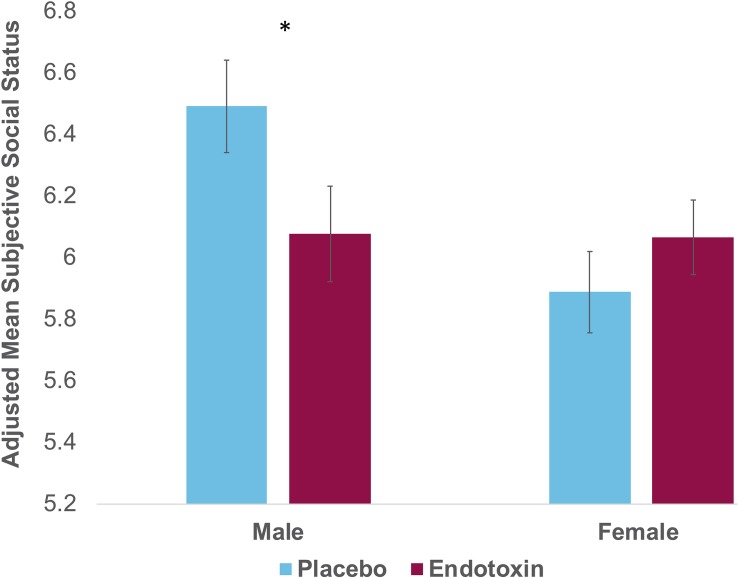
Self-reported scores on the MacArthur Scale of Subjective Social Status at *T2* (peak of inflammatory response for the endotoxin group) as a function of condition (endotoxin vs. placebo) and sex. Plotted values reflect estimated marginal means from the condition^∗^sex analysis; displayed values and statistical analyses adjusted for *T0* values of subjective social status, and for feelings of social disconnection and depression at *T2.* Error bars depict the standard error of the adjusted mean.

Next, we examined the effect of sex and condition on subjective social status. There was no main effect of condition [F(1,107) = 0.69, *p* = 0.41]; there was a main effect of sex [F(1,107) = 4.88, *p* = *0.03*], such that males show higher social status than females overall. Interestingly, as hypothesized, we did find a significant sex by condition interaction [Figure 1; F(1,107) = 4.36, *p* = 0.04; η_p_^2^ = 0.04]. Follow-up analyses revealed that there were significant condition effects for males [F(1,40) = 4.50, *p* = 0.04; η_p_^2^ = 0.10]. As depicted in Figure 1, males exposed to endotoxin reported lower subjective social status at *T2* relative to males in the placebo condition. This condition effect was not significant for females [F(1,64) = 3.01, *p* = 0.09], indicating no difference in subjective social status between females exposed to endotoxin vs. placebo. These results indicate significant effects of endotoxin (vs. placebo) on subjective status for males, but not for females.

### Relationships Between Cytokines and Subjective Social Status Scores

For those within the endotoxin group (collapsed across males and females), there was no significant correlation between subjective social status scores and IL-6 or TNF-α (*p*’s > 0.2). Within males separately, there was no significant correlation between subjective social status scores and IL-6 or TNF-α (*p*’s > 0.6). There was also no significant correlation for females (*p*’s > 0.2).

## Discussion

The present findings extend the literature on inflammation and social experience by examining, for the first time, the effects of an experimental inflammatory challenge on subjective social status. Consistent with our hypothesis, we found that males exposed to endotoxin, relative to males exposed to placebo, showed lower levels of subjective social status. This effect was not present in females. Thus, these results indicate that males may be more sensitive to inflammation-induced changes in the domain of social status.

Why would males be more likely to report feeling lower in subjective social status as a result of being exposed to endotoxin? Although this question has never been directly tested in previous studies, these findings do fit with other results in the literature. First, correlational work has found that the relationship between subjective social status and inflammation is stronger in men than women (Freeman et al., 2016), which complements our finding of lower perceived social status among men exposed to an inflammatory challenge. Further, at a biological level, testosterone may also help explain sex differences in the link between inflammation and social status. Testosterone, which is found in higher concentrations in men than women, is associated with social behaviors known to be involved in the “achievement and maintenance of social status” (Shattuck and Muehlenbein, 2015a). Indeed, men’s social status (vs. women’s) is more consistently linked with testosterone (Booth et al., 2006). Furthermore, testosterone is related to inflammation and sickness behavior; testosterone typically decreases in response to sickness, including decreasing in response to an experimental inflammatory challenge in humans (Shattuck and Muehlenbein, 2015a, b). Thus, it is possible that decreases in testosterone in men exposed to endotoxin, as occurs during sickness, may underlie their feelings of lower subjective social status compared to men in the placebo group. This idea will need to be tested explicitly in a future study, as no testosterone measures were taken in the current study.

In addition to biological underpinnings, the psychological concept of subjective social status may also be particularly important for males relative to females. Males’ self-esteem, more than females’ self-esteem, is more deeply rooted in their social status (Fischer and Mosquera, 2001), and males’ social status vs. females’ social status is more relevant to their ability to secure a mate (Buss, 1989; Zentner and Mitura, 2012; Freeman et al., 2016). As such, subjective social status may be a more self-relevant or sensitive concept for males vs. females. Interestingly, in a qualitative analysis of the MacArthur Scale of Subjective Social Status (i.e., the measure of subjective social status used in the present study), participants often noted that they used “being respected” as a criteria for where they placed themselves on the ladder (Adler and Stewart, 2007). Given that males may be particularly sensitive to feeling the loss of respect of others (Fischer and Mosquera, 2001), males may be more sensitive than females to decreasing in subjective social status as a result of feeling vulnerable during sickness.

The sex differences we observed here, with men showing increased sensitivity to the effects of inflammation on one aspect of social experience (i.e., subjective social status), are in contrast to previous sex differences observed by our group and others in other domains of social experience (Eisenberger et al., 2009; Moieni et al., 2015c; Lasselin et al., 2018). For example, we have previously found that females showed greater increases in feelings of social disconnection in response to endotoxin (Moieni et al., 2015c). However, we have also shown that there are no sex differences in decreases in social cognitive abilities in response to endotoxin (Moieni et al., 2015b). Taken together with the results of the present study, these findings indicate that females are not uniformly more sensitive to all inflammatory-induced changes in social experience. Indeed, this suggests that a nuanced approach in which multiple domains of social experience (e.g., both social connection and perceived social status) are assessed is necessary to understand sex differences in the context of inflammation and social behavior.

This study is not without its limitations. First, the sample was relatively young and healthy, and the results may look different in older and/or clinical samples. Indeed, there is some evidence from non-human animal models that behavioral effects of experimental inflammation could differ by age (Kinoshita et al., 2009). Furthermore, although we suspect that decreases in testosterone could underlie the lower levels of subjective social status observed for endotoxin males, we did not directly measure testosterone. Future studies could measure testosterone and test whether decreases in testosterone mediate changes in subjective social status, particularly for males. Furthermore, although this measure is not influenced by shifts in negative mood (Kraus et al., 2013), we controlled for depressed mood and feelings of social disconnection in all analyses to be conservative and further ensure that effects in the present paper were not simply duplications of previous findings from this same sample (Moieni et al., 2015c). Additionally, we did not find significant relationships between changes in subjective social status and cytokines among subjects in the endotoxin group, including no significant correlations when examining males and females separately. It is possible there is no dose-dependent relationship between cytokines and changes in subjective social status; it is also possible that this specific measure of subjective social status is not sensitive enough to directly correlate with changes in cytokines. Furthermore, ovulatory cycles and hormones may play an important role for females, and we did not collect information about cycles in our dataset in order to examine this as a factor; future research should consider this important factor for sex differences in inflammatory-induced changes. Finally, the analyses in this paper reflect a secondary analysis of a previous dataset; the power analyses for the current study were calculated for the primary aims of the previous dataset. As such, it is possible we were underpowered to detect the specific effects of interest in this paper.

In summary, this study contributes to the literature by examining sex differences in subjective social status in response to experimental inflammation. Males exposed to endotoxin, vs. males exposed to placebo, showed significantly lower subjective social status, but this was not the case for females. This sex difference in sensitivity to subjective social status builds on sex differences found in the literature more broadly and suggests that a nuanced study of sex differences across a variety of social experiences is necessary in order to fully understand changes in social behavior due to inflammation.

## Data Availability Statement

The raw data supporting the conclusions are available by request by contacting the corresponding author, NE, at neisenbe@ucla.edu. Data will be made available in this fashion because we did not ask our human subjects ahead of time if we could share the data. We will therefore make data (stripped of subjects’ identifying information) available upon request to be conservative toward protecting subjects.

## Ethics Statement

This study, involving human participants, was reviewed and approved by the UCLA Human Subjects Protection Committee. The participants provided their written informed consent to participate in this study.

## Author Contributions

NE and MI were responsible for the overall study’s concept and design. KM was responsible for conceptualizing the social status piece of the design. IJ was the study coordinator and responsible for running the experimental sessions. MM was responsible for statistical analyses. EB was responsible for the performance, review, and quality control of all cytokine assays. MM and NE drafted the manuscript. All authors approved the final manuscript.

## Conflict of Interest

The authors declare that the research was conducted in the absence of any commercial or financial relationships that could be construed as a potential conflict of interest.
