# Supplementary material for: Sex Differences in the Effect of Inflammation on Subjective Social Status: A Randomized Controlled Trial of Endotoxin in Healthy Young Adults
Source: Front Psychol. 2019 Oct 1;10:2167. doi: 10.3389/fpsyg.2019.02167 (PMC6781934; doi:10.3389/fpsyg.2019.02167)
Supplement: Supplementary file 1 [file Table_1.DOC]

**CONSORT DIAGRAM**

Randomized (n= 123)

**Allocation**

**Analysis**

**Enrollment**

Assessed for eligibility (n= 2116)

Excluded (n= 1993)

♦  Not meeting inclusion criteria (n= 1967)

♦  Declined to participate (n= 15)

♦  Other reasons (n= 11)

Analysed (n= 54)

Allocated to placebo condition (n= 57)

♦ Received placebo (n= 54)

♦ Did not receive placebo (n= 3)

subject cancelled (n= 3)

Allocated to endotoxin condition (n= 66)

♦ Received endotoxin (n= 61)

♦ Did not receive endotoxin (n= 5)

subject cancelled (n= 1)

difficulty drawing blood (n= 1)

lost consciousness prior to receiving drug (n= 1)

failed drug test prior to session (n= 2)

Analysed (n= 61)
