# Supplementary material for: Sex Differences in the Effect of Inflammation on Subjective Social Status: A Randomized Controlled Trial of Endotoxin in Healthy Young Adults
Source: Front Psychol. 2019 Oct 1;10:2167. doi: 10.3389/fpsyg.2019.02167 (PMC6781934; doi:10.3389/fpsyg.2019.02167)
Supplement: Supplementary file 2 [file Table_2.DOCX]

**Subjective Social Status Scores**

These scores reflect Descriptive Statistics of Subjective Social Status at Baseline and T2 by Condition and Sex. These are presented as Mean (SD).

|  | **Placebo Male** | **Endotoxin Male** | **Placebo Female** | **Endotoxin Female** |
| --- | --- | --- | --- | --- |
| **Baseline** | 6.00 (1.68) | 6.87 (1.52) | 5.90 (1.78) | 6.42 (1.46) |
| **T2** | 6.30 (1.61) | 6.57 (1.50) | 5.71 (1.72) | 6.03 (1.57) |
